# Thermal-Feature System Identification for a Machine Tool Spindle

**DOI:** 10.3390/s19051209

**Published:** 2019-03-09

**Authors:** Yuh-Chung Hu, Ping-Jung Chen, Pei-Zen Chang

**Affiliations:** 1 Department of Mechanical and Electro-Mechanical Engineering, National ILan University, Yilan 26047, Taiwan; 2 Institute of Applied Mechanics, National Taiwan University, Taipei 10617, Taiwan; r05543089@ntu.edu.tw (P.-J.C.); changpz@ntu.edu.tw (P.-Z.C.)

**Keywords:** machine learning, machine tool spindle, system identification, temperature sensor, thermal feature model

## Abstract

The internal temperature is an important index for the prevention and maintenance of a spindle. However, the temperature inside the spindle is undetectable directly because there is no space to embed a temperature sensor, and drilling holes will reduce its mechanical stiffness. Therefore, it is worthwhile understanding the thermal-feature of a spindle. This article presents a methodology to identify the thermal-feature model of an externally driven spindle. The methodology contains self-made hardware of the temperature sensing and wireless transmission module (TSWTM) and software for the system identification (SID); the TSWTM acquires the temperature training data, while the SID identifies the parameters of the thermal-feature model of the spindle. Then the resulting thermal-feature model is written into the firmware of the TSWTM to give it the capability of accurately calculating the internal temperature of the spindle from its surface temperature during the operation, or predicting its temperature at various speeds. The thermal-feature of the externally driven spindle is modeled by a linearly time-invariant state-space model whose parameters are identified by the SID, which integrates the command “n4sid” provided by the System ID Toolbox of MATLAB and the *k*-fold cross-validation that is common in machine learning. The present SID can effectively strike a balance between the bias and variance of the model, such that both under-fitting and over-fitting can be avoided. The resulting thermal-feature model can not only predict the temperature of the spindle rotating at various speeds but can also calculate the internal temperature of the spindle from its surface temperature. Its validation accuracy is higher than 98.5%. This article illustrates the feasibility of accurately calculating the internal temperature (undetectable directly) of the spindle from its surface temperature (detectable directly).

## 1. Introduction

High-speed machining is a promising technology to dramatically increase productivity and reduce costs. Most machining errors in machine tools come from thermal problems [1,2]. During high-speed machining of a spindle, a lack of complete information of its temperature variation may lead to an abrupt malfunction due to overheating. Therefore, it is very important to monitor and predict the temperature variation of the spindle during the machining process. In order to accurately monitor the temperature inside the spindle, the authors propose a methodology, which combines hardware and software into an edge-computing module, to identify the thermal-feature of the spindle. One can accurately monitor the temperature inside the spindle from its surface temperature through the proposed module. Research on the thermal analysis of spindles has been going on for quite a long time. Bossmanns and Tu [3,4] developed the thermal and power flow models of a motorized spindle based on a finite difference method (FDM). Many studies [5,6,7] have adopted a finite element method to simulate the thermal behavior, such as temperature variation and thermal deformation, of machine tool spindles. Brecher et al. [8] developed a thermal-resistance network model of an externally driven spindle based on its structural and heat transfer properties and pointed out that the main source of heat generation is bearing. The author, Hu, [9] developed a speed-dependent thermal network model of a spindle based on the principle of heat transfer and empirical formulae. Most complex systems can be modeled based on experimental data and system identification methods even without physical insight, therefore system identification methods have been widely used in various fields for prediction and control. Cho [10] modeled an active magnetic bearing system with a discrete-time state-space model and estimated the parameters of the model with a system identification method based on input and output data. Eguia et al. [11] exerted a subspace system identification method to model the thermal behavior of multicore microprocessors for temperature estimation. Skibinski and Sethares [12] used a recursive identification method to analyze the thermal impedance of a semiconductor package. On the other hand, wireless real-time temperature monitoring facilitates the inspection and protection of machines [13,14,15]. Experiments [16,17] have shown that the resistance temperature detector (RTD) is ideal for temperature sensing because of its linearity, accuracy, long-term stability, and wide-range of temperature measurement.

Among the published literature, research on the thermal-mechanical behavior of machine tool spindles mainly used the finite difference method, finite element method, thermal and power flow model, thermal resistance network model, etc. This paper uses the state-space model to simulate the thermal-mechanical behavior of machine tool spindles. The novelty of this paper is the use of the n4sid command provided by the system identification toolbox of MATLAB and the k-fold cross validation in machine learning to estimate the state-space model of machine tool spindles from measured input and output data. The present method can effectively strike a balance between the bias and variance of the model, such that both under-fitting and over-fitting can be avoid. This article presents a methodology to identify the thermo-feature of the spindle. The methodology contains hardware, the temperature sensing and wireless transmission module (TSWTM), and software, the thermal-feature system identification (SID) for the spindle. The TSWTM acquires the temperature training data, while the SID identifies the parameters of the thermo-feature model of spindle. The TSWTM is equipped with seven RTDs to acquire the temperature training data from seven characteristic positions of the spindle, where five are interior to it and two are located on its surface. The input data of the thermo-feature model of a spindle is the time series of rotational speed or the temperatures on the surface of the spindle, and the observation/output data are the temperatures at the characteristic positions of the spindle. Both are entered into the SID to identify the parameters of the thermo-feature model of the spindle. The resulting thermo-feature model accurately predicts the temperature variation inside or on the surface of the spindle.

## 2. Methodology

### 2.1. Temperature Sensor and Wireless Transmission Module (TSWTM)

**Figure 1:**
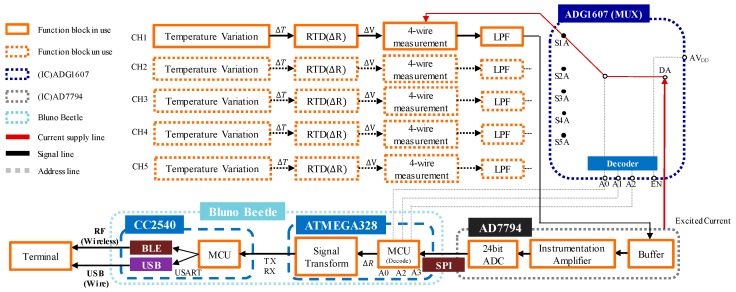
Functional block diagram of the temperature sensor and wireless transmission module (TSWTM).

**Figure 2:**
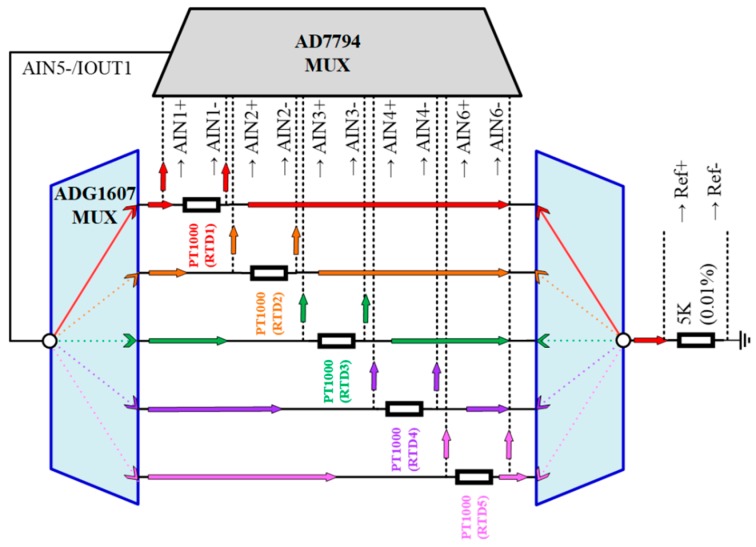
Schematic diagram of the current flow path for an analog input channel in parallel.

The TSWTM is composed of 5 RTDs, a multiplexer (ADG1607), an Analog-to-Digital Converter (AD7794), and an Arduino Bluno Beetle board with Bluetooth 4.0 (Figure 1). The RTDs (Pt-1000) are used to detect the temperature variations of the spindle’s five feature-points based on the 4-wires measurement. The ADG 1607 [18] (Figure 2) switches on one of the 5 RTDs each time according to the command decided by the micro controller unit (ATMEGA328) through the three-bit address lines (A0, A1, A2) (Figure 1). The AD7794 [19] (Figure 2) is configured for 6 differential input channels corresponding to the 5 RTDs and a reference resistance to implement the 4-wires measurement of the RTDs’ cross voltages. As shown in Figure 1, after the low-pass filtering (LPF), the cross voltage of the RTD will be transmitted into AD7794 through the differential analog input channel and transformed to a digital signal and then received by ATMEGA328 through the ICSP port. The digitalized voltage signal of the RTD is transferred into a temperature signal by the signal transform firmware. Afterward, the temperature signal is received by the CC2540 through the transceiver (TX) and receiver (RX) pins. Finally, the temperature signal is transmitted to the terminal through Bluetooth or USB.

**Figure 3:**
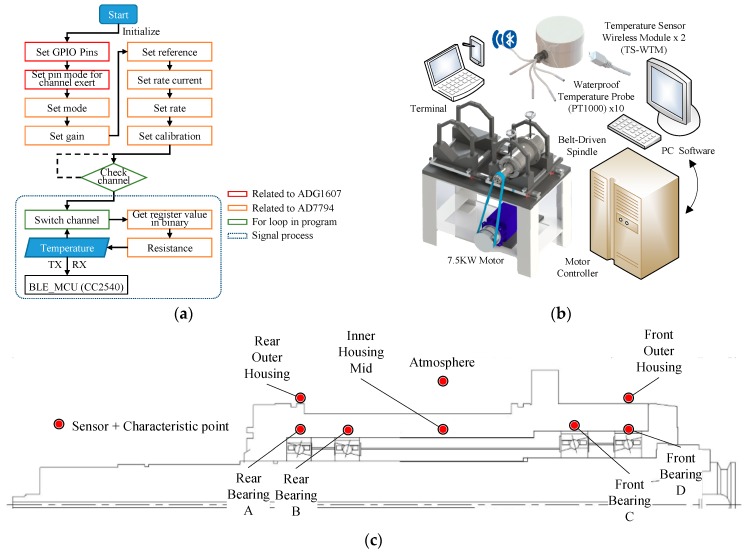
(**a**) Signal transform firmware flow chart; (**b**) Experiment setup schematic; (**c**) Sensor location and characteristic points on the spindle.

The firmware used to transform the digitalized voltage signals of RTDs into temperature signals is programmed with Arduino. Figure 3a details the flowchart of the signal transform firmware. First, the pins of A0, A2, and A3 on the Bluno Beetle are defined as the general-purpose output (GPO) pins, they output a high or low potential according to the truth table (Table 1) set up in the register. The analog-to-digital converter (ADC) is initially set up with the external reference, gain of two, excitation current of 210 µA, calibration, 4.17 Hz update rate, and continuous read mode. The channel in connection would be identified according to the examination of the resistance value before the signal process stage. After the initialization stage, the micro control unit (MCU) receives and converts the register number of the analog input voltage into binary code to calculate the resistance of the RTD based on Equation (1) provided by the AD7794 datasheet.

(1) $$\mathrm{Code}=2^{N}\times [(AIN\times GAIN/V_{ref})+1].$$

The temperature can be determined by the relationship of temperature and resistance given by Equation (2) from the DIN EN 60751 standard which defines Pt100 resistance accuracy classes and corresponding tolerance,

(2) $$R(T)=R_{0}(1+AT+BT^{2})$$

where

$$A=3.9083\times 10^{-3}\;{}^{\circ}C^{-1};\;B=-5.775\times 10^{-7}\;{}^{\circ}C^{-2}.$$

The actual temperature could be transmitted to the Bluetooth-MCU (BLE-MCU) (CC2540) through the transceiver (TX) and receiver (RX) pins.

### 2.2. Experiment Setup

#### 2.2.1. Spindle Run-In System

**Figure 4:**
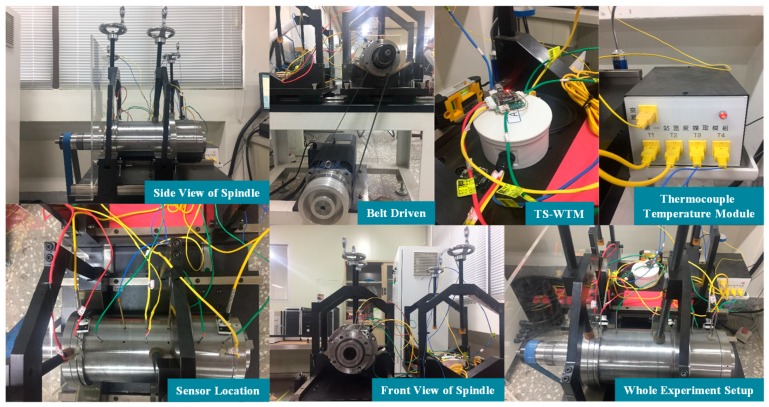
The photos of the experiment setup.

**Figure 5:**
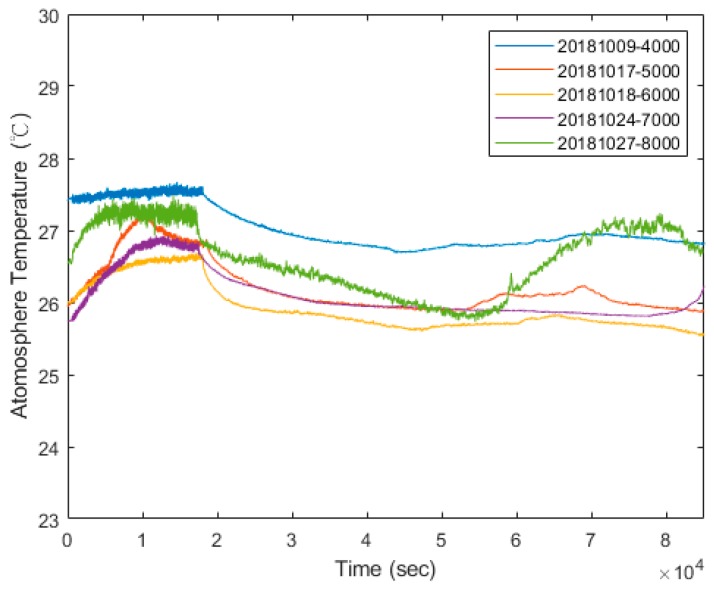
The variation of ambient temperature during a day.

The run-in system of the spindle, shown in Figure 3b, consists of a spindle externally driven by a 7.5 kW motor through a belt, a controller accompanied by a personal computer (PC), used to control the motor through a programmable control interface, and two sets of self-made TSWTMs with temperature RTD (PT1000) sensor probes. As shown in Figure 3c, RTDs are located at the outer rings of front and rear bearings, the center point of the spindle’s inner housing, the rear and front ends of the spindle’s outer housing surface, and the ambient temperature to acquire the training data for the thermal feature identification algorithm. Figure 4 shows the photos of the experimental setup. Each run of experiments lasts for almost a day; therefore, the variation in ambient temperature during the day (Figure 5) must be considered. Based on the literature survey in Introduction, one knows that bearings are the main heat source of spindles, therefore the bearings are important temperature characteristic points and choosing the outer rings of front and rear bearings to set the RTD is important because they are static rings. As conduction dominates the heat transfer of the metallic inner housing and is very fast, one must choose the center point of the inner housing because it is the same distance away from either end. In fact, spindles do not allow holes drilled in them to set the RTD when in use, therefore one must choose the rear and front ends of the outer housing surface to set the RTD because they are the only positions being capable of detecting temperature directly when in use.

#### 2.2.2. Performance of the Temperature Sensing and Wireless Transmission Module (TSWTM)

**Figure 6:**
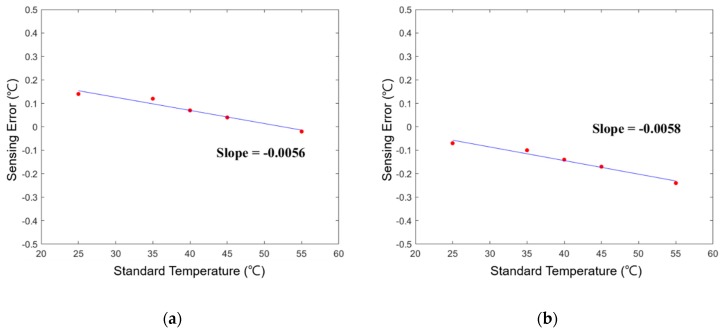
The sensing error of the TSWTM at various temperatures: (**a**) CH1; (**b**) CH2.

**Figure 7:**
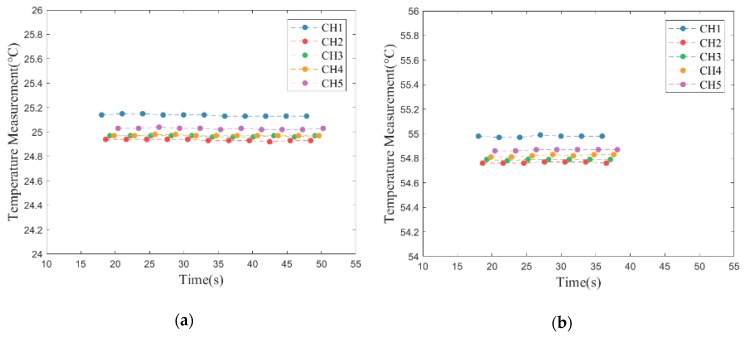
Temperature measurement of the TSWTM elapsing a time-period at constant temperature: (**a**) 25 °C; (**b**) 55 °C.

**Figure 8:**
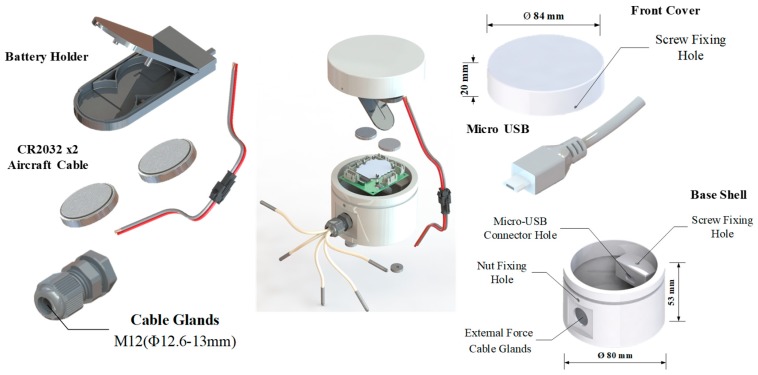
Introduction of all components used in the TSWTM.

The performance of the self-made TSWTM was tested in a precisely temperature-controlled environment provided by the Electronics Testing Center (ETC) in Taiwan. The constant-temperature environment is controlled by liquid calibration baths (LCB) and the standard temperature is measured by a high-accuracy (@–100 to 100 °C ± 0.009 °C) platinum RTD thermometer [20]. Given the results of channels 1 and 2 of the TSWTM as examples, Figure 6a,b show their sensing error over the temperature range of 25 to 55 °C. The sensing error at different temperatures can be fitted with a first-order linear equation. The slope of error to temperature of channels 1 to 5 are −0.0056, −0.0058, −0.0061, −0.0051, and −0.0054 respectively, which demonstrate that the five channels of TSWTM are consistent with each other. Therefore, the differences in sensing error between the five channels are negligible. The ordinates of Figure 7a,b are the temperature measured by the TSWTM at the standard temperature of 25 and 55 °C, respectively, while their abscissas are the elapsed times, which demonstrate the high stability and good anti-interference ability of the TSWTM. Table 2 and Table 3 list the specifications of the TSWTM and temperature probe, respectively. The bare size of the TSWTM is 43 × 48 × 10 mm^3^ and the package size is Ø 80 × 53 mm^3^. The TSWTM measures the temperature with a temperature probe (PT1000, Ø3 probe). Its measurement range is −50 to 300 °C, the accuracy is @ 25 ± 0.05 °C, and the power consumption is 175 mW. Figure 8 shows the components in use and assembly process of the TSWTM.

### 2.3. System Identification for the Thermal-Feature Model of the Spindle

**Figure 9:**
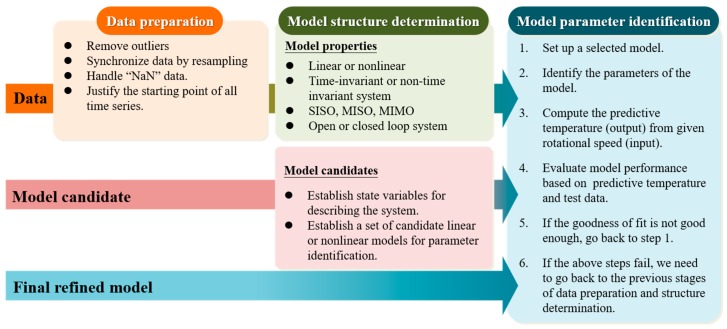
Procedure of thermo-feature identification.

During the operation of the spindle, the temperature variations within it involves complex heat transfer phenomena and unpredictable environmental disturbances. As a result, it is difficult to analyze its heat transfer phenomena by means of theoretical approaches [21]. On the other hand, SID is a method for building the mathematical model of a system from the data measured during its operation [22]. The authors model the thermal-feature of an externally driven spindle in terms of a state-space representation based on the input rotational speed and the output temperature datasets measured during its operation. The process of SID for the thermal-feature model of the spindle can be roughly divided into three stages: data preparation, model structure determination, and model parameter identification, as shown in Figure 9. The three stages are explained in the following:

1. Data preparation: This stage prepares the dataset for SID. More specifically, this stage has to remove the outliers, perform resampling, remove missing data, and justify the starting time for all the time-series sequences.
2. Model structure determination: This stage is for deciding several important model properties, such as linear or nonlinear, and time-invariant or time-varying, and then determine the model structure, namely the types and corresponding structure configurations accordingly.
3. Model parameter identification: This stage is for identifying the best parameters of the thermal-feature model based on the measured input/output data of the spindle in operation. After the identification, the resulting thermal-feature model is validated by the test data to verify whether the model’s prediction is accurate enough. If the accuracy is not good enough, one has to change the method of parameter identification. Furthermore, once the change of the parameter identification method cannot generate satisfactory accuracy, one has to go back to the previous stage to re-determine the model structure. In fact, the stages of model structure determination and parameter identification are often repetitively interleaved until the model with the best structure and parameters is found. It should be mentioned that the test data used to validate the model is always different from the training data used to identify the model.

#### 2.3.1. Data Preparation Stage

**Figure 10:**
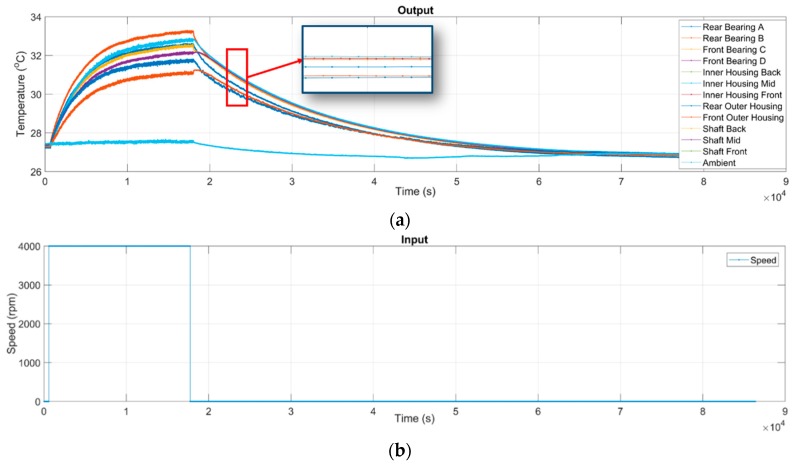
Raw data: (**a**) The time-series of temperatures measured by the TSWTM; (**b**) the time-series of input rotational speed of spindle. Given the case of 4000 rpm as an example.

Recall that the input and output data of the thermal-feature model of the spindle are its rotational speeds and temperatures, respectively. The rotational speed of the spindle is controlled by a programmable control interface, while the temperatures are measured by the self-made TSWTM. The spindle was set to rotate at a certain constant speed, meanwhile its temperature variation was recorded continuously. The spindle was turned down to stationary if the temperature variation was less than 1 °C for 3 consecutive hours. The temperature was recorded continuously until the spindle naturally cooled to ambient temperature. The whole process lasted a full day for each specific speed of the spindle, the records are detailed in Table 4. Figure 10 shows the raw data of 4000 rpm as an example.

As the temperatures at the different locations in and on the spindle are acquired by different channels of the TSWTM, the temperature sampling timings of different locations are not synchronous. The center symbols shown in the zoom-in window of Figure 10 demonstrate that the temperature raw data acquired by different channels are in different sampling times. For system identification, the raw data has to be cleaned and synchronized through the following procedure:

1. Plot the raw data for visual inspection. If there some extremely erroneous data exists, which may be due to device failure or some unknown reasons, then consider such data to be an outlier and remove it all directly.
2. As the sampling timings of all channels are nonsynchronous, it is necessary to resample the all-time series of output temperatures and input speeds to reach the same sample rate of 1 Hz.
3. The data of not a number (NaN) has to be removed from the time series because it represents missing-data. Furthermore, the all-time series should start simultaneously with the first time sample that has all non-NaN temperatures.

**Figure 11:**
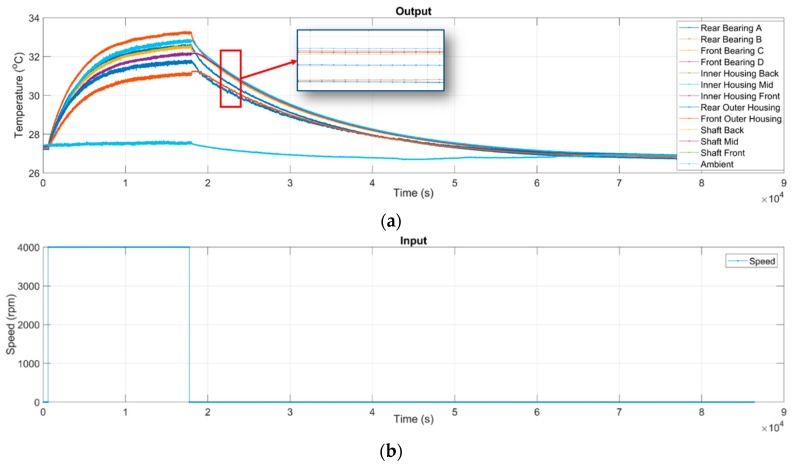
Resampled data with the same sampling rate of 1 Hz: (**a**) The time-series of temperatures resampled from the raw data; (**b**) The time-series of the input rotational speed of the spindle resampled from the raw data. Given the case of 4000 rpm as an example.

**Figure 12:**
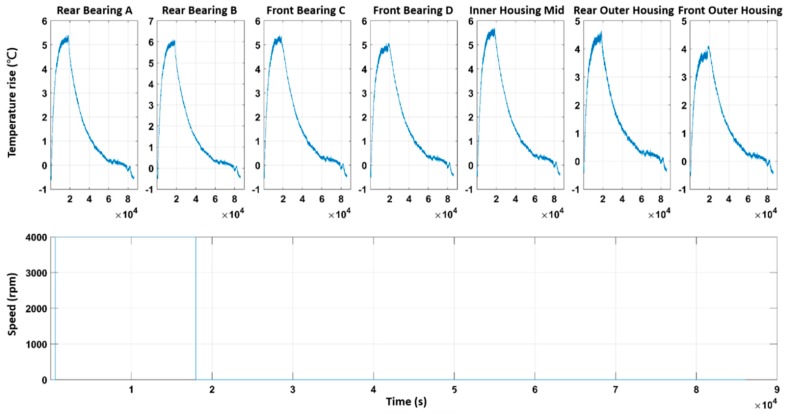
The final dataset for system identification. Given the case of 4000 rpm as an example.

Given the spindle speed of 4000 rpm as an example, Figure 10 shows the measured raw data, wherein each temperature curve has a different and possibly uneven sampling rate. Figure 11 demonstrates that the data resampled from the raw data have the same sample rate of 1 Hz. For system identification, one still needs to remove the NaN data and subtract the ambient temperature from the measured temperature data, and adjust the starting time. The final data ready for system identification is shown in Figure 12, wherein the rotational speed and the temperature serve as the input and output data, respectively, for system identification.

#### 2.3.2. Structure Determination Stage

**Figure 13:**
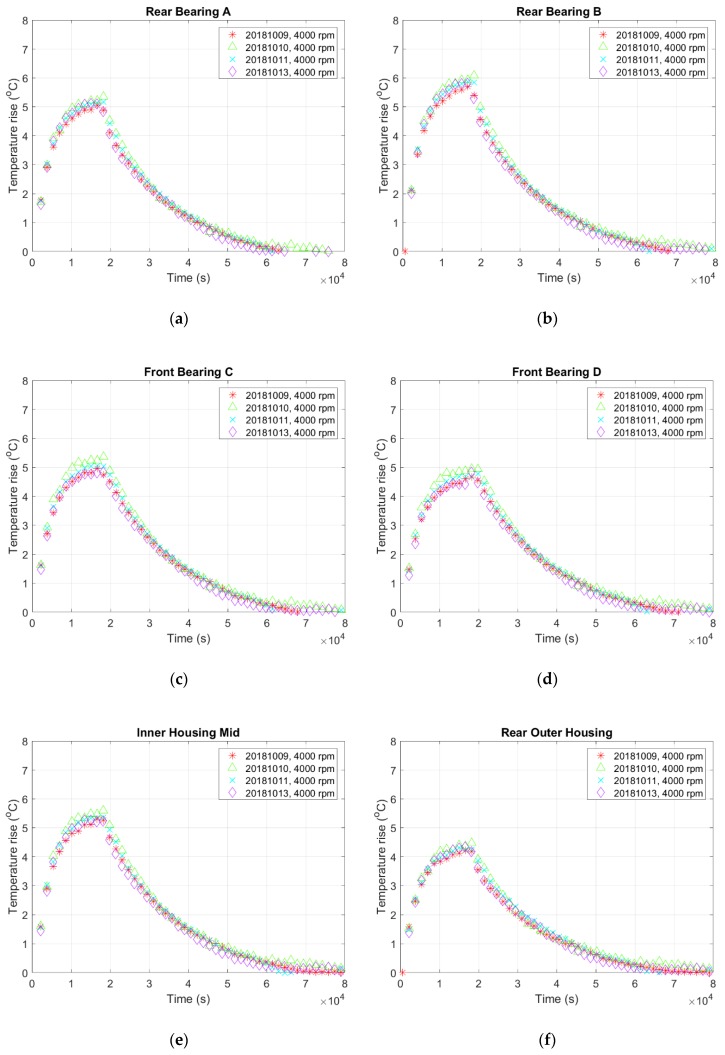

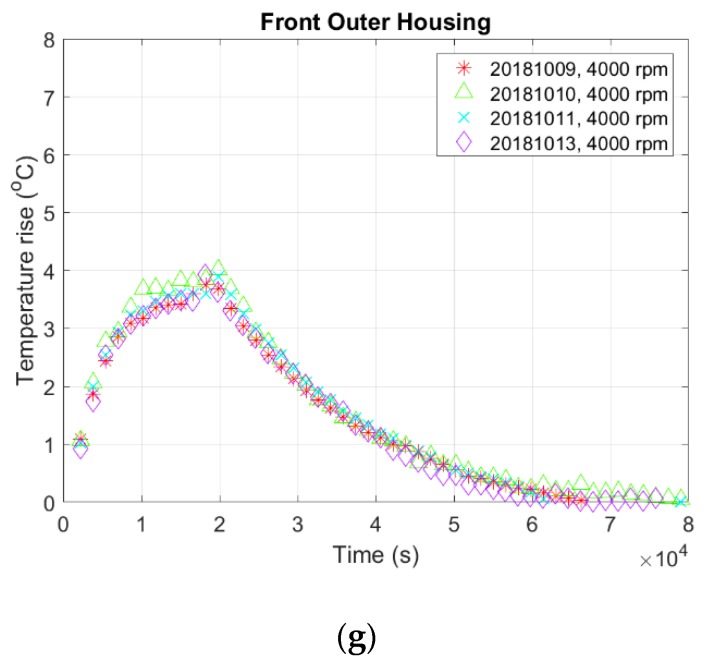
The temperature variations of seven spindle’s characteristic points at the same rotational speeds to that of Figure 8b but measured on different dates: (**a**) rear bearing A; (**b**) rear bearing B; (**c**) front bearing C; (**d**) front bearing D; (**e**) inner housing mid; (**f**) rear outer housing; (**g**) front outer housing.

When a system is too complicated to be modeled based on a theoretical approach, one can model it mathematically based on the correlation between input and output data. For the thermal-feature model of the spindle, the input is its rotational speed while the output is its temperature variation measured by the TSWTM. Conceptually, a model can be classified into linear or nonlinear and time-invariant or time-varying. Figure 13 shows the temperature variations of seven spindle’s characteristic points at the same rotational speeds to that of Figure 10b, which were measured on different dates. These figures reveal that the temperature progressions do not change much on different dates and thereby demonstrate the time-invariance of the thermal-feature model of the spindle. According to modern control theory, a dynamic system can be represented by a state-space model [23], which is usually sufficient to describe a dynamic system accurately [24]. Therefore, the authors chose a linearly time-invariant state-space model provided by the System ID Toolbox of MATLAB [24] to model the thermal-feature of the spindle in the following parameter identification stage.

#### 2.3.3. Parameter Identification Stage

According to modern control theory [23], the linear time-invariant model for modeling a physical system can be expressed in terms of the state-space equation

(3) $$\{\begin{array}{ccc} \frac{x(t+\Delta t)-x(t)}{\Delta t} & = & \mathrm{Ax}(t)+\mathrm{Bu}(t)+\mathrm{Ke}(t),\;x(0)=x_{0},\\ y(t) & = & \mathrm{Cx}(t)+e(t). \end{array}$$

where **u**(*t*) is a vector of input data whose components are the time-series of all input data, **y**(*t*) is a vector of output data whose components are the time-series of all output data, **e**(*t*) is the vector of noise signals that cannot be reduced during the system identification process, **x**(*t*) is the vector of state variables whose dimension is dependent on the number of order (*n*) of the model, and the entries of matrices, **A**, **B**, **C**, **D**, and **K**, are the parameters of the model to be identified to fit the given training data during the identification process and whose dimensions are dependent on the number of order of the model as well as the number of input/output data. For the case of the spindle in this article, the input data is only its time-series of rotational speed (single input), while the output data are the time-series of temperature variations of its seven characteristic points (7 outputs) shown in Figure 3c. Therefore, **u**(*t*) is a 1-dimensional vector whose component is the time-series of rotational speed, **y**(*t*) is a 7-dimensional vector whose components are the time-series of the temperature variations of the 7 characteristic points, and **e**(*t*) is a 7-dimensional vector.

**Figure 14:**
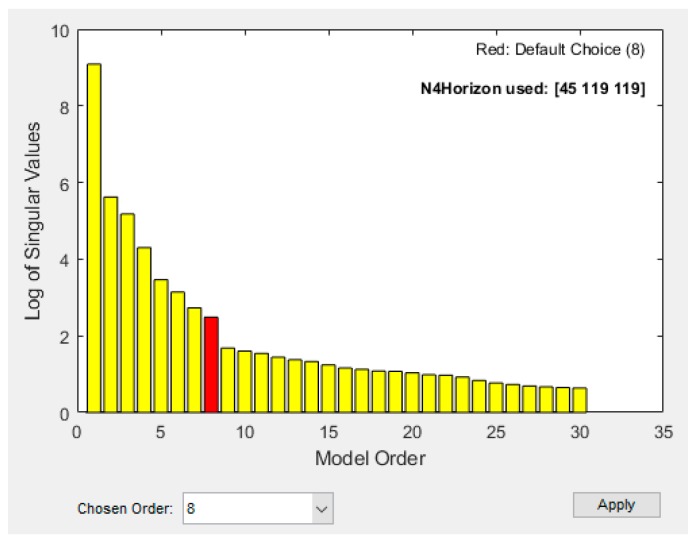
Select the model order by the command “n4sid” in the System ID Toolbox of MATLAB.

There are several commands, such as n4sid, ssest, and ssregest, in the System ID Toolbox of MATLAB for system identification [24]. As the first one, n4sid, is the fastest compared with the others, the authors use it throughout this article. The training data, i.e., the fold 1 of Table 4, was put into the command n4sid to conduct the system identification process. The command n4sid allows one to set a range of the model order (*n*) and determine the best model order within this range by the Hankel singular value. The Hankel singular values measure the contribution of each state to the input/output behavior, namely the states with small Hankel singular values can be discarded to simplify the model. As a result, n4sid determines the best model order to be 8, as shown in Figure 14. Therefore, with the 8-order state space model, the state space equation of the spindle is expressed as

(4) $$\begin{array}{ccc} \left\{\begin{array}{c} x_{1}(t+1)\\ x_{2}(t+1)\\ \vdots \\ x_{8}(t+1) \end{array}\right\} & = & \left[\begin{array}{cccc} a_{1,1} & a_{1,2} & \cdots & a_{1,8}\\ a_{2,1} & a_{2,2} & \cdots & a_{2,8}\\ \vdots & \vdots & \ddots & \vdots \\ a_{8,1} & a_{8,2} & \cdots & a_{8,8} \end{array}\right]\left\{\begin{array}{c} x_{1}(t)\\ x_{2}(t)\\ \vdots \\ x_{8}(t) \end{array}\right\}+\left\{\begin{array}{c} b_{1}\\ b_{2}\\ \vdots \\ b_{8} \end{array}\right\}u(t)+\left[\begin{array}{cccc} k_{1,1} & k_{1,2} & \cdots & k_{1,7}\\ k_{2,1} & k_{2,2} & \cdots & k_{2,7}\\ \vdots & \vdots & \ddots & \vdots \\ k_{8,1} & k_{8,2} & \cdots & k_{8,7} \end{array}\right]\left\{\begin{array}{c} e_{1}(t)\\ e_{2}(t)\\ \vdots \\ e_{7}(t) \end{array}\right\},\\ \left\{\begin{array}{c} y_{1}(t)\\ y_{2}(t)\\ \vdots \\ y_{7}(t) \end{array}\right\} & = & \left[\begin{array}{cccc} c_{1,1} & c_{1,2} & \cdots & c_{1,8}\\ c_{2,1} & c_{2,2} & \cdots & c_{2,8}\\ \vdots & \vdots & \ddots & \vdots \\ c_{7,1} & c_{7,2} & \cdots & c_{7,8} \end{array}\right]\left\{\begin{array}{c} x_{1}(t)\\ x_{2}(t)\\ \vdots \\ x_{8}(t) \end{array}\right\}+\left\{\begin{array}{c} d_{1}\\ d_{2}\\ \vdots \\ d_{8} \end{array}\right\}u(t)+\left\{\begin{array}{c} e_{1}(t)\\ e_{2}(t)\\ \vdots \\ e_{7}(t) \end{array}\right\}. \end{array}$$

After determination of the best model order and the parameters identification of the corresponding model, the predictive temperature and goodness of fit of the model can be evaluated by invoking the command “compare,” which will be discussed in the following section, Results and Discussion.

Instead of using the variation in Hankel singular value (as used in “n4sid” of MATLAB), the authors propose another way to determine the best model order by means of *k*-fold cross-validation, which is commonly used in machine learning [25]. For simplicity, the authors adopt 2-fold cross-validation with varying model order to determine what order is the best one that leads to the highest validation accuracy (goodness of fit). The criterion of goodness of fit of each output is defined by the normalized root mean square error (*NRMSE*),

(5) $$NRMSE_{i}=1-\frac{\|X_{obs}(:,i)-X(:,i)\|}{\|X_{obs}(:,i)-mean(X_{obs}(:,i))\|},$$

where *X_obs_*(:,*i*) and *X*(:,*i*) are respectively the observed and predicted time-series of *i*-th output and *mean*( ) is the mean value of a time-series of data. Then the performance of SID is evaluated by the mean value of *NRMSE_i_*, that is

(6) $$MNRMSE=\frac{1}{N}\sum_{i=1}^{N}NRMSE_{i}.$$

where *N* is the number of outputs and *N* = 7 for this article because there are 7 characteristic temperature points in/on the spindle. Let $M_{rpm,n}^{fold}$ be the *n*-order model identified from the training data $D_{rpm}^{fold}$ listed in Table 4, then its overall accuracy can be evaluated by the *MNRMSE*, namely the *X*(:,*i*) is the time-series predicted by $M_{rpm,n}^{fold}$ and *X_obs_*(:,*i*) is a given data $D_{rpm}^{fold}$. For convenience of explanation, in the following, express the overall accuracy of the model evaluated by the aforesaid *MNRMSE* by

(7) $$Accuracy=test(M_{rpm,n}^{fold},D_{rpm}^{fold}).$$

If the given data $D_{rpm}^{fold}$ is the same as the training data of $M_{rpm,n}^{fold}$, then the accuracy is called the training accuracy (*TA*) otherwise its called the validation accuracy (*VA*). For a given order *n*, one can evaluate the overall training accuracy (*TA*) by

(8) $$TA=\frac{1}{2}[test(M_{rpm,n}^{1},D_{rpm}^{1})+test(M_{rpm,n}^{2},D_{rpm}^{2})].$$

Similarly, the overall validation accuracy (*VA*) is evaluated by

(9) $$VA=\frac{1}{2}[test(M_{rpm,n}^{1},D_{rpm}^{2})+test(M_{rpm,n}^{2},D_{rpm}^{1})].$$

Based on the concept of cross-validation, which is commonly used in machine learning for classification and regression [26], it is necessary to vary the model complexity, namely the model order *n*, and search for a n* that maximizes the validation accuracy,

(10) $$n^{*}=\arg\underset{n}{\max}\;VA(rpm,n).$$

**Figure 15:**
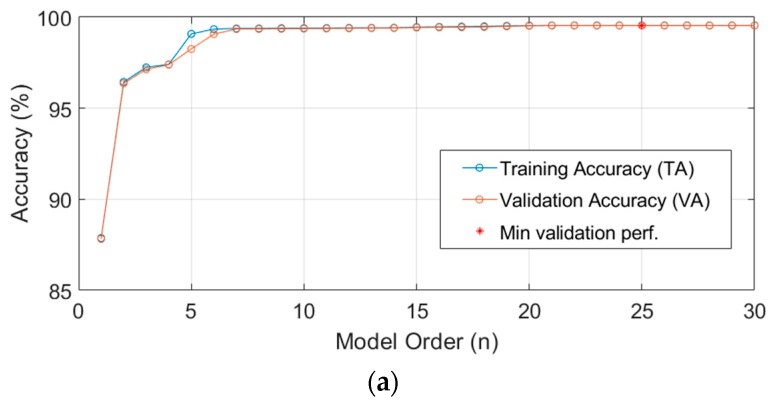

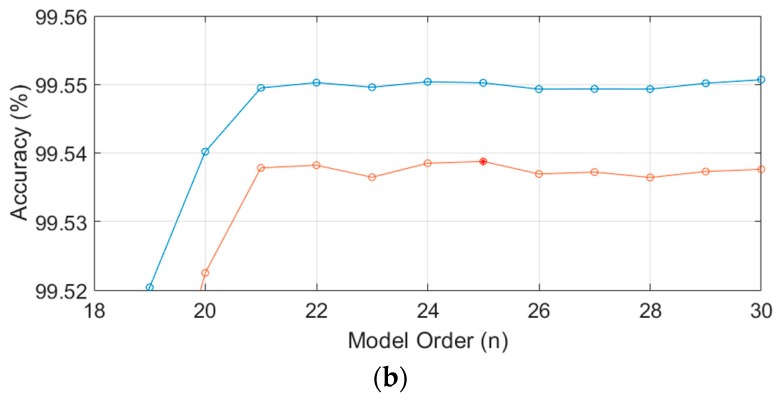
Model order determination via 2-fold cross validation: (**a**) The training and validation accuracies by *MNRMSE* at different model orders, wherein the best model order of 25 is indicated by an asterisk; (**b**) Zoom-in to the range near order 25.

Using cross-validation to determine the optimal complexity of a model can effectively strike a balance between bias and variance, such that both under-fitting and over-fitting can be avoided. Table 5 lists all the results of 2-fold cross-validation for the model at given rotational speeds as mentioned above, which shows that the model order of 25 results in the best *MNRMSE* in validation accuracy. As an example, Figure 15 shows the goodness of fit (*MNRMSE*) of the model, which is trained by the data $D_{5000}^{1}$ and tested with the data $D_{5000}^{2}$.

## 3. Results and Discussions

### 3.1. Speed-Dependence of the Thermal-Feature Model of the Spindle

**Figure 16:**
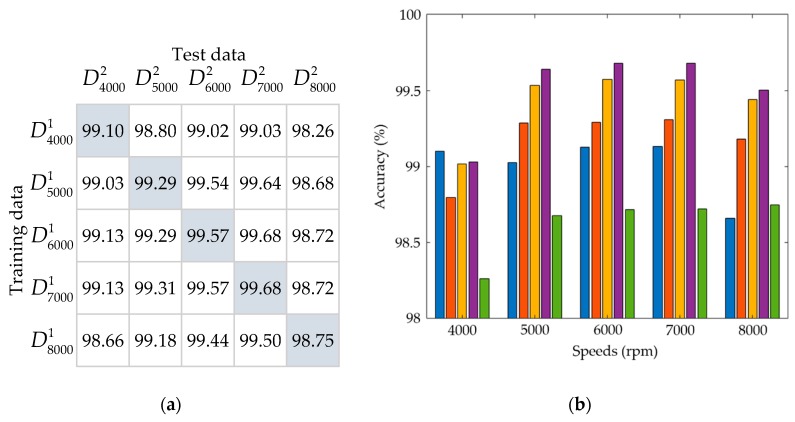
The percentage VA of the 8-order model: (**a**) The matrix chart; (**b**) The bar chart, wherein each group of bars corresponds to a row of the matrix chart.

**Figure 17:**
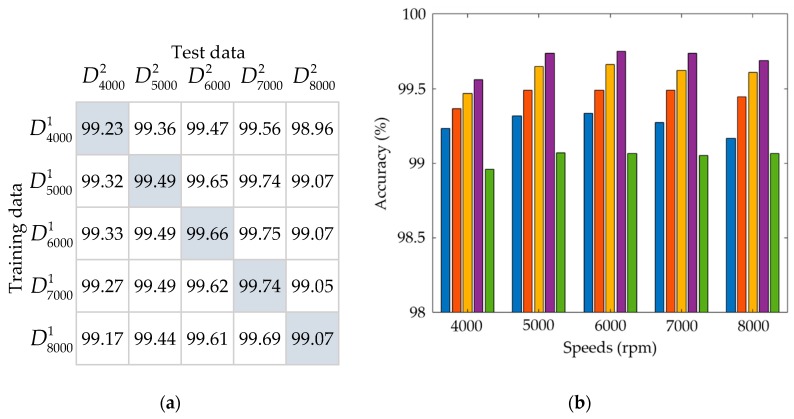
The percentage VA of the 25-order model: (**a**) The matrix chart; (**b**) The bar chart, wherein each group of bars corresponds to a row of the matrix chart.

As the thermal-feature model of the spindle is identified by the correlation between the input data, i.e., the time-series of spindle speed, and the output data, i.e., the time-series of spindle temperature at the input spindle speed, then, for different input spindle speeds, the identified parameters of thermal-feature models are slightly different. Hence it is worthwhile investigating the influence of the spindle speed on the variance of the parameters of the thermal-feature model. Here the authors use the data in the fold 1 of Table 4 as the training data to identify the model and those in the fold 2 as the test data to test the validation accuracies of each model. The results are displayed in both a matrix form and a bar chart as shown in Figure 16 and Figure 17, wherein the former is identified by an 8-order model and the latter by a 25-order model. In the matrix forms, Figure 16a and Figure 17a, the entry (i, j) represents the percentage VA of the model using the training data $D_{rpm_{i}}^{1}$ and test data $D_{rpm_{j}}^{2}$. Each row entry of the matrix forms are also displayed in bar charts, Figure 16b and Figure 17b, corresponding to a specific spindle speed. If the model is highly dependent on the spindle speed, then the VAs of the diagonal entries in the matrix forms should be much higher than the other entries. However, as shown in the bar charts, this is not true. The VAs of all models are very close, for 8- and 25-order models, they are about 98.5% and 99%, respectively. Therefore, the influence of the spindle speed on the variance of the model is approximately negligible. This means that one may use the thermal-feature model identified at a certain spindle speed to predict the spindle temperature at other speeds. It is rational to assume the thermal-feature model of the spindle is linear and time-invariant.

### 3.2. Using the Thermal-Feature Model to Predict the Temperature Variation of the Spindle at Various Speeds

**Figure 18:**
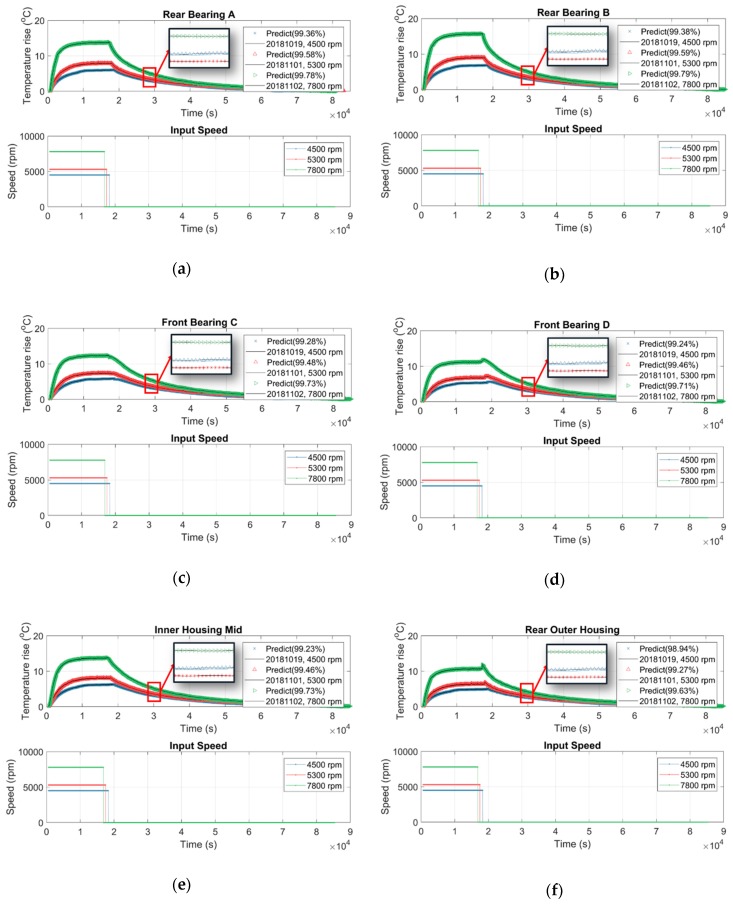

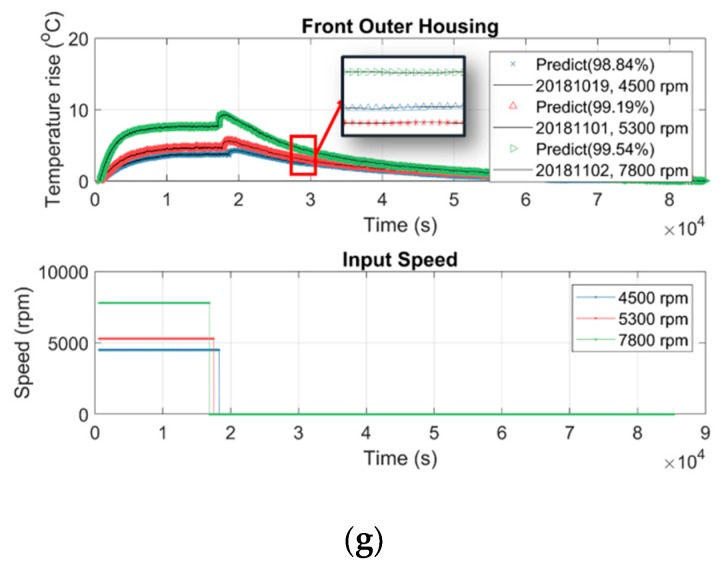
Using the thermal-feature model of the spindle to predict the temperature variation at: (**a**) rear bearing A; (**b**) rear bearing B; (**c**) front bearing C; (**d**) front bearing D; (**e**) inner housing mid; (**f**) rear outer housing, (**g**) front outer housing.

The previous section concluded that the influence of spindle speed on the variance of the thermal-feature model is approximately negligible, namely the parameters of the thermal-feature model are constant. This section tries to use the thermal-feature model $M_{6000,8}^{1}$, namely the 8-order model identified from the training data $D_{6000}^{1}$ listed in Table 4 to predict the temperature variation of the spindle rotating at various speeds. The spindle was set to rotate at a certain constant speed, meanwhile its temperature variation was recorded continuously. The spindle was turned down to stationary if its temperature variation was less than 1 °C for 3 consecutive hours. The temperature was recorded continuously until the spindle naturally cooled to ambient temperature. Figure 18 shows the test results of the rotational speeds of 4500, 5300 and 7800 rpm, respectively, where the zoom-in window clearly illustrates that the prediction is very close to the measured data, the goodness of fit (MNRMSE) is over 98.94% for all locations. These results confirm that the thermal-feature model of the spindle can be represented by a linearly time-invariant state-space model with constant parameters.

### 3.3. Predicting Internal Temperature of the Spindle from Its Surface Temperature

**Figure 19:**
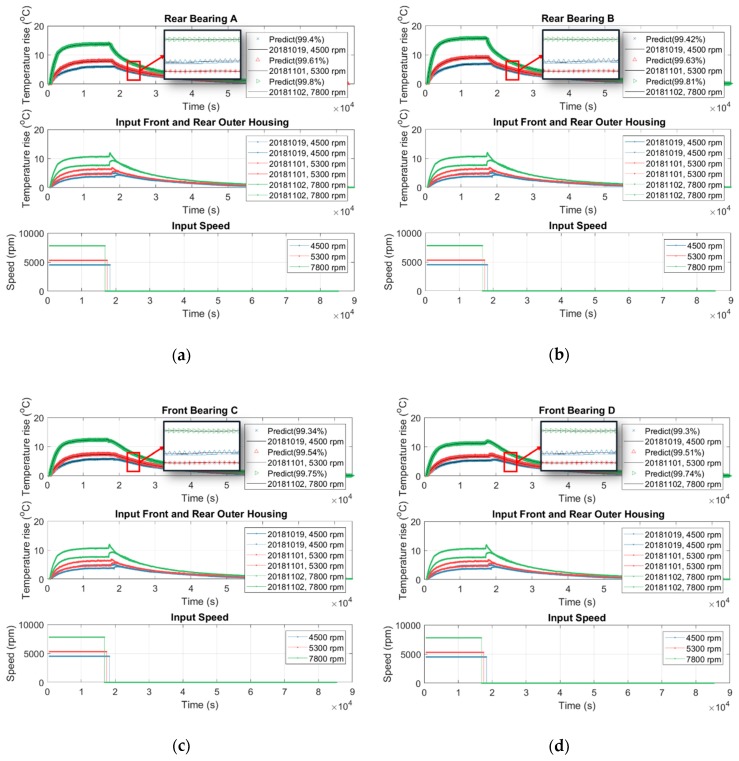

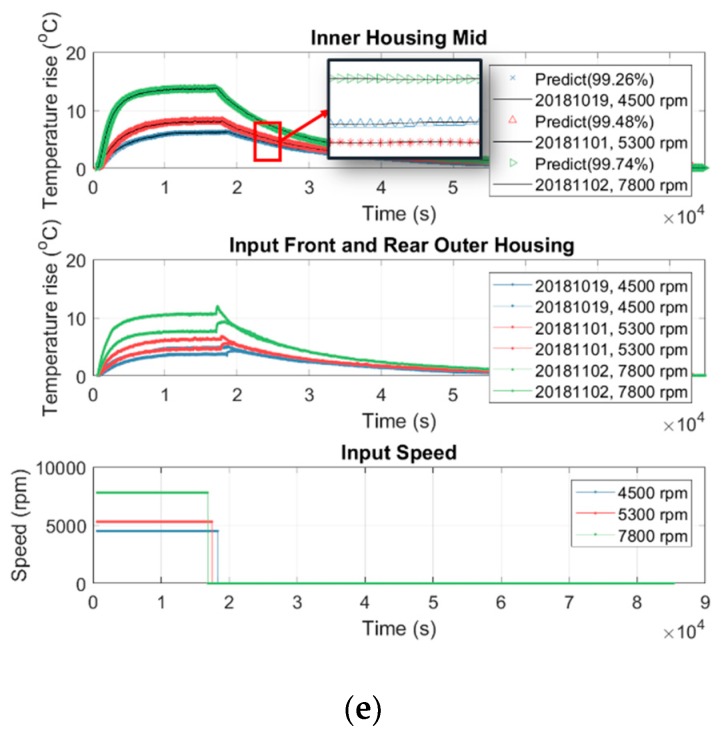
Using the 3I5O thermal-feature model (Table 6) to predict the internal temperature of the spindle at: (**a**) rear bearing A; (**b**) rear bearing B; (**c**) front bearing C; (**d**) front bearing D; (**e**) inner housing mid.

**Figure 20:**
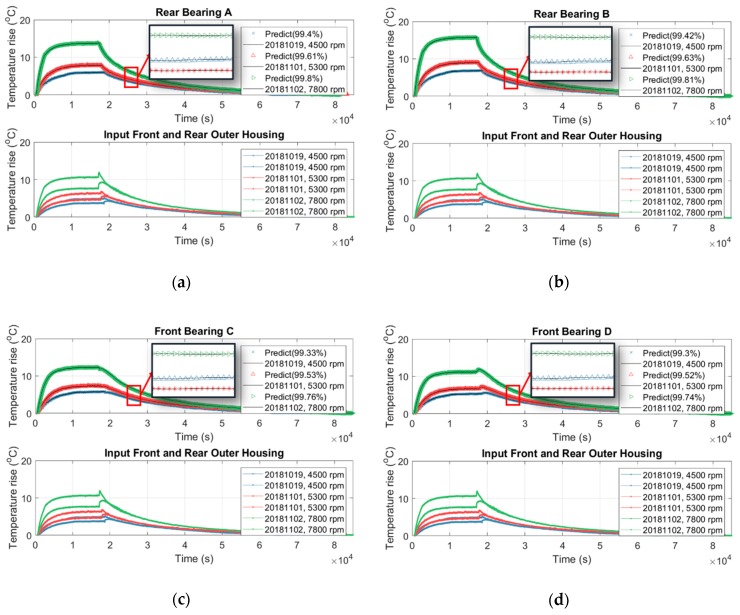

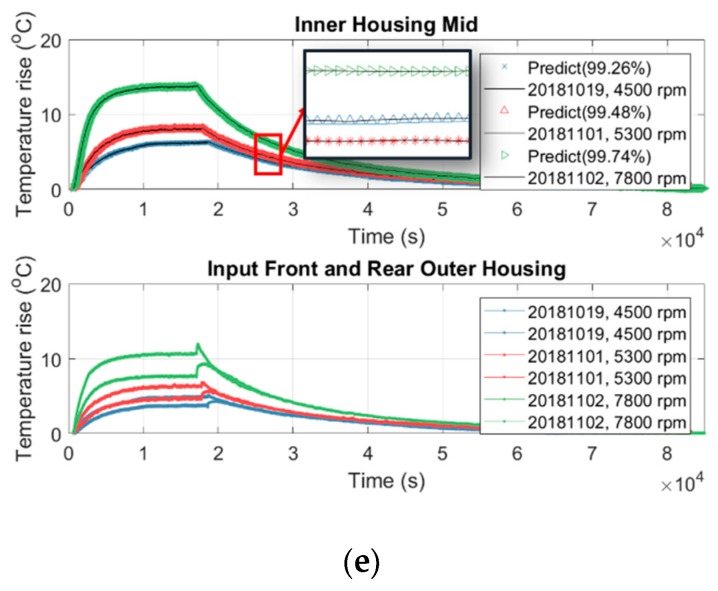
Using the 2I5O thermal-feature model (Table 6) to predict the internal temperature of the spindle at: (**a**) rear bearing A; (**b**) rear bearing B; (**c**) front bearing C; (**d**) front bearing D; (**e**) inner housing mid.

**Figure 21:**
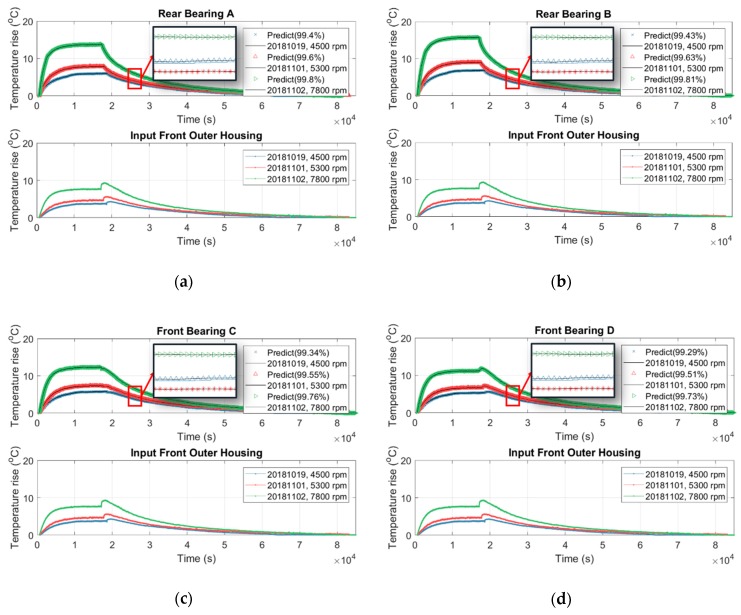

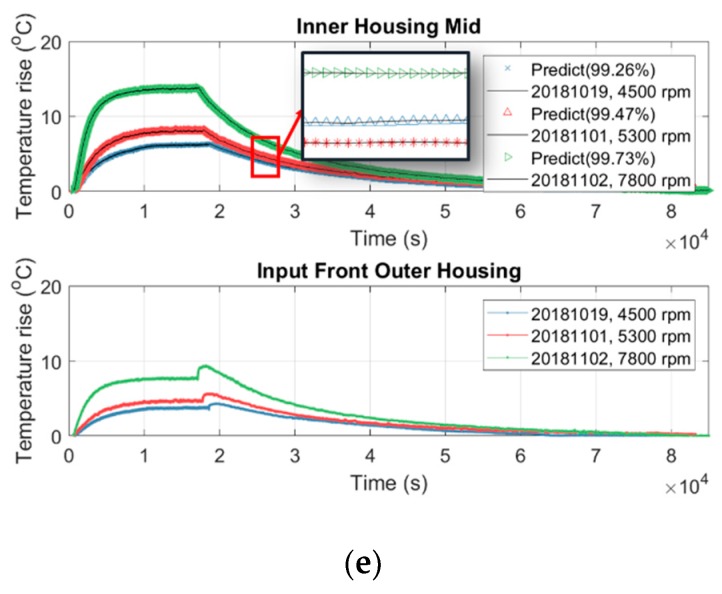
Using the 1I5O thermal-feature model (Table 6) to predict the internal temperature of the spindle at: (**a**) rear bearing A; (**b**) rear bearing B; (**c**) front bearing C; (**d**) front bearing D; (**e**) inner housing mid.

**Figure 22:**
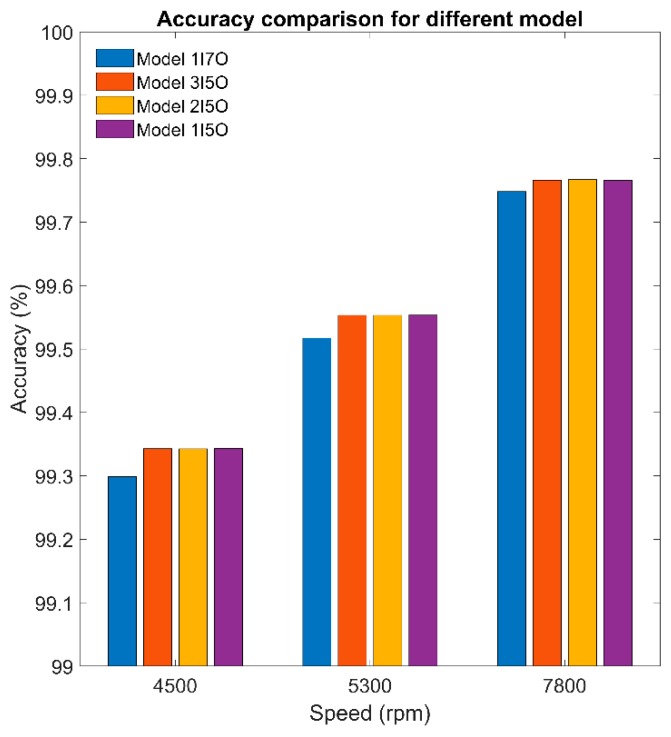
The accuracies of temperature prediction by the thermal-feature models listed in Table 6.

In fact, the temperature inside the spindle is undetectable because there is no space inside the spindle to embed the temperature sensor. In addition, the spindle manufacturer or user never allows drilling on the spindle to place the temperature sensor inside because these holes may reduce the mechanical stiffness of the spindle. However, the internal temperature may provide an important index for prevention and maintenance of the spindle. Section 3.1 and Section 3.2 conclude that the speed-dependence of the thermal-feature model of the spindle is approximately negligible and it can be represented by a linearly time-invariant state-space model with constant parameters. Therefore, this section tries to use the temperature on the surface of the outer housing of the spindle, which is detectable directly, as the input data to identify the thermal-feature model of the spindle, and then, by the resulting thermal-feature model, to figure out the undetectable temperature inside the spindle from its surface temperature. We set the spindle to rotate at a constant speed of 6000 rpm, meanwhile we recorded its temperature variation continuously. The spindle was turned down to stationary if its temperature variation was less than 1 °C for 3 consecutive hours. The temperature was recorded continuously until the spindle naturally cooled to ambient temperature. The time-series of temperature of seven points inside/outside the spindle, as shown in Figure 3c, are recorded and serve as input or output data for the identification of the thermal-feature model, described in Table 6. According to the number of input (I) and output (O) data, four thermal-feature models of the spindle, namely 1I7O, 3I5O, 2I5O, and 1I5O, are respectively identified by the linearly time-invariant 8-order state-space model. Then the resulting thermal-feature models were used to predict the temperature of the spindle at various speeds, say 4500, 5300, and 7800 rpm. Figure 16 shows the results predicted by the 1I7O model, while Figure 19, Figure 20 and Figure 21 show the results predicted by the models of 3I5O, 2I5O, and 1I5O, respectively. The prediction results agree very well with the measured data; as shown in Figure 22, whose accuracies are over 99%. This illustrates the feasibility of predicting the internal temperature of the spindle from its surface temperature.

## 4. Conclusions

This article proposes a methodology for the thermal-feature system identification of a machine tool spindle. A self-made temperature sensing and wireless transmission module was developed to acquire the training data required for system identification, with an accuracy of ±0.05 °C at 25 °C, measurement range −50 to 300 °C, 5 support channels, sampling rate 4.17 Hz, power consumption 175 mW, and protection level IP65. The thermal-feature of the spindle was modeled by a linearly time-invariant state-space model. The parameters of the state-space model are identified by the command “n4sid” provided by the System ID Toolbox of MATLAB. The best model-order was doubly checked by the Hankel singular value (used in “n4sid” of MATLAB) and *k*-fold cross validation (common in machine learning), wherein the accuracy of an 8-order model is about 98.5%, while that of a 25-order model is about 99%. The 25-order model is the optimal model for the present case. However, for the calculation efficiency, the 8-order model is a good model for the present case. The proposed method to determine the optimal complexity of a model, namely model order, can effectively strike a balance between bias and variance, such that both under-fitting and over-fitting can be avoided. Section 3.1 illustrates that the influence of spindle speed on the variance of the thermal-feature model of the spindle is approximately negligible. It is rational to assume the thermal-feature model of the spindle is linearly time-invariant. Section 3.2 illustrates that the thermal-feature model of the spindle can be represented by a linearly time-invariant state-space model with constant parameters. This means that one may use the thermal-feature model identified at a certain spindle speed to predict the spindle temperature at other speeds. Based on the assumption that the spindle speed has a negligible effect on the variance of the thermal-feature model, Section 3.3 uses the temperature on the surface of the outer housing of the spindle as input data and its internal temperature as output data to identify its thermal-feature model. Section 3.3 illustrates the feasibility of predicting the internal temperature (undetectable directly) of the spindle from its surface temperature (detectable directly). The internal temperature may provide an important index for prevention and maintenance of the spindle. It should be mentioned here that the research objective of this article is an externally driven spindle. However, for a motorized spindle, its thermal-feature model might be highly dependent on the spindle speed, which is worthy of studying in the near future. The applicability of the present methodology is not limited to externally-driven spindles, although it is validated by the run-in test of our available externally-driven spindle. Based on the results of the run-in test, it is indeed accurate to simulate the externally-driven spindle by a state-space model with constant coefficients. However, based on the literature, the state-space model of motorized spindles ought to use non-constant coefficients. For example, Lin and Tu [27] had mentioned that high-speed rotation can cause substantial changes in the dynamic and thermal behaviors of motorized spindles, and Liu and Zhang [28] concluded that the electromagnetic power loss of the built-in motor with motor slip at high-speed rotation will change the thermal-mechanical properties of motorized spindles.

## Figures and Tables

**Table 1:** Part of the ADG1607 truth table [18].

A2	A1	A0	EN	Switch
0	0	0	1	1
0	0	1	1	2
0	1	0	1	3
0	1	1	1	4
1	0	0	1	5

**Table 2:** Specification of TSWTM.

**Accuracy [**°C**]**	@25 ± 0.05
**Measurement Range [**°C**]**	−50 to 300
**Supportable Channels**	5
**Sampling Rate [Hz]**	4.17
**Power Consumption [mW]**	175
**Size [mm^3^]**	Ø 80 × 53
**Communication**	BLE/Micro USB
**Power Supply**	Wire (USB)/Wireless (CR2032)

**Table 3:** Specifications of the temperature sensing probe.

**Sensor Type**	PT1000 (A class)
**Accuracy [**°C**]**	±(0.15 ± 0.002|t|)
**Measurement Range [**°C**]**	−50 to 300
**Excited Current Limit [mA]**	≤5
**Thermal Response [s]**	≤0.3 @ air
**Size [mm^3^]**	Ø 3 × 60
**Package Material**	Stainless steel 304
**Protection Level**	IP 65

**Table 4:** The data descriptions for thermal-feature model identification, all data were recorded in the year 2018. The superscript of the data name indicates the fold number and the subscript indicates the input spindle speed. The format of the recorded date is month/day. Note that each data contain 1 time-series of input spindle speed and 7 time-series of temperature variation corresponding to 7 characteristic temperature points of the spindle.

Input Speed [rpm]		4000	5000	6000	7000	8000
**Fold 1**	**Data**	$D_{4000}^{1}$	$D_{5000}^{1}$	$D_{6000}^{1}$	$D_{7000}^{1}$	$D_{8000}^{1}$
	**Record Date**	10/09	10/17	10/18	10/24	10/28
**Fold 2**	**Data**	$D_{4000}^{2}$	$D_{5000}^{2}$	$D_{6000}^{2}$	$D_{7000}^{2}$	$D_{8000}^{2}$
	**Record Date**	10/10	10/29	10/30	10/25	10/27

**Table 5:** Results of 2-fold cross validation at a given rotational speeds.

Rotational Speed [rpm]	4000	5000	6000	7000	8000
Best Model Order (n*)	25	25	26	25	21
**Training Accuracy (TA) [%]**	99.34	99.55	99.71	99.73	99.17
**Validation Accuracy (VA) [%]**	99.23	99.54	99.69	99.7	99.16
**Elapsed Time [sec]**	11231.9	10871.3	10692.5	10816.1	9784.31

**Table 6:** The thermal-feature models and their input/output data.

I/O No.	Input Data	Output Data
1I7O	1. Spindle speed	1. Temperature of rear bearing A 2. Temperature of rear bearing B 3. Temperature of front bearing C 4. Temperature of front bearing D 5. Temperature of inner housing 6. Temperature of front outer housing 7. Temperature of rear outer housing
3I5O	1. Spindle speed 2. Temperature of front outer-housing 3. Temperature of rear outer-housing	1. Temperature of rear bearing A 2. Temperature of rear bearing B 3. Temperature of front bearing C 4. Temperature of front bearing D 5. Temperature of inner housing
2I5O	1. Temperature of front outer-housing 2. Temperature of rear outer-housing	
1I5O	1. Temperature of front outer-housing	
